# Comparative Study on the Hydration, Mechanical Properties, and Energy Storage Performance of MPC-Based Solid Electrolytes Modified by Different Ionic PAMs

**DOI:** 10.3390/ma19071426

**Published:** 2026-04-02

**Authors:** Jialu Liu, Yunpeng Zhang, Muyang Shi, Xin Shan, Dong Zhang

**Affiliations:** 1 Key Laboratory of Advanced Civil Engineering Materials, Ministry of Education, School of Materials Science and Engineering, Tongji University, Shanghai 201804, China; 2331576@tongji.edu.cn (J.L.); smyovo@126.com (M.S.); 2 China Construction Eighth Engineering Division Co., Ltd., Shanghai 200112, China; zh_yunpeng@126.com

**Keywords:** magnesium phosphate cement, polyacrylamides, ionic types, structural supercapacitors, electrochemical properties

## Abstract

**Highlights:**

- The first study on MPC electrolyte modified by three different ionic types of polyacrylamides (PAMs) was conducted.
- Elucidated the distinct behaviors and mechanisms of three different ionic types of PAMs in regulating the microstructure and properties of MPC.
- The optimal synergy of mechanical and electrochemical properties was achieved using a 0.5% dosage of anionic PAM, exhibiting favorable cost-effectiveness and superior performance.

**Abstract:**

Polyacrylamides (PAMs) exhibit variable molecular characteristics, exerting different effects on the macro- and micro-properties of cement when used as cement modifiers. In this study, three different ionic polyacrylamides, namely anionic PAM (APAM), cationic PAM (CPAM), and nonionic PAM (NPAM), were employed to investigate their impacts on the microstructure, hydration, mechanical properties, and electrochemical energy storage characteristics of magnesium phosphate cement (MPC). Microstructural characterization methods, including Mercury Intrusion Porosimetry (MIP), Scanning Electron Microscopy (SEM), Fourier transform infrared (FTIR), etc., were employed to elucidate the phase composition, micromorphology, and pore structure of the modified MPC. Furthermore, electrochemical testing methods such as Cyclic Voltammetry (CV), Galvanostatic Charge–Discharge (GCD), and Electrochemical Impedance Spectroscopy (EIS) were conducted to reveal the energy storage characteristics of supercapacitors. The results indicate that all three PAMs retarded the hydration of MPC, optimized the pore size distribution, and increased the compressive strength of MPC. In terms of electrochemical performance, the ionic conductivity of MPC electrolyte modified by NPAM and APAM exhibited an opposite trend to that of CPAM as the dosage increased. At dosages of 0.5% (NPAM), 1.5% (CPAM), and 0.75% (APAM), the assembled supercapacitors using PAM-modified MPC electrolyte exhibited excellent areal capacitance and energy density, reaching a maximum areal capacitance of 1060 mF cm^−2^ and an energy density of 0.147 mWh cm^−2^.

## 1. Introduction

The built environment is associated with considerable material and energy consumption, contributing approximately 23% of total CO_2_ emissions from global economic activities [1,2,3]. To mitigate building energy consumption, renewable energy has been increasingly adopted. However, due to the inherent intermittency of such energy sources, large-scale green energy storage buildings have emerged as a promising solution. Traditional batteries are constrained by high costs, safety issues, and short cycle life, rendering them unsuitable for large-scale applications in renewable energy storage [4,5]. Cement-based structural supercapacitors (CSSCs) offer the advantages of cost-effectiveness, durability, and structural compatibility, enabling their potential wide application in residential energy storage, road charging, and structural health monitoring [6,7,8]. CSSCs represent an important development direction for energy-storage buildings and are anticipated to become an optimal choice for large-scale energy storage systems [9,10,11,12].

CSSCs were first proposed by Zhang et al. [12], who successfully prepared and studied CSSCs by integrating cementitious materials with energy storage capabilities. Early research primarily focused on Portland cement systems. Those studies have revealed that modified Portland cement electrolytes exhibit excellent mechanical properties, achieving a compressive strength of up to 40 MPa. Simultaneously, the assembled supercapacitors maintain favorable electrochemical performance with an areal capacitance reaching 1.4 F cm^−2^. However, current Portland cement-based systems still suffer from low ionic conductivity and limited energy density. For instance, Zhan et al. [13] used steel slag powder-modified Portland cement to prepare electrolytes, but the electrolytes had the problem of low ionic conductivity (10 mS cm^−1^). To tackle this challenge, researchers turned to the advanced magnesium phosphate cement (MPC) system, known for its superior properties such as rapid hardening, high strength, micro-expansion, and superior bonding strength [14,15,16,17]. Its main hydration product, potassium struvite (K-struvite), provides a stable crystalline basis for enhancing energy storage performance [18]. The graphene-MPC supercapacitor proposed by Ma et al. [19] achieved a maximum specific capacitance of 46.38 F g^−1^, confirming its energy storage potential. However, the problem of “mechanical-electrochemical conflict” has been observed, where electrochemical performance often declines as compressive strength increases. To optimize the synergy between these properties, various polymers, such as Polyvinyl Alcohol (PVA), Polyacrylic Acid (PAA), and Polyethylene Oxide (PEO), have been introduced to modify cement electrolytes [20,21,22,23]. As a modifier, PAM shows significant potential in cementitious systems due to its ability to optimize pore structure and construct conductive networks for efficient ion transport [24,25,26]. Jiao et al. [26] modified MPC electrolytes with anionic PAM via in situ polymerization, achieving a specific capacitance of 32 F g^−1^ and an ionic conductivity of 54 mS cm^−1^ while maintaining a compressive strength of 30.2 MPa. This validated the synergistic enhancement effect of polymers.

Currently, research on energy storage in PAM-modified cementitious materials has primarily focused on the anionic PAM, with significant advancements in energy storage. However, PAM is categorized into three primary types: cationic (CPAM), which contains quaternary ammonium groups; anionic (APAM), characterized by carboxyl groups; and non-ionic (NPAM), featuring amide groups in their side-chain functional groups. Significant differences exist in the performance regulation of cement modified by different ionic PAMs [27,28]. For example, Yuan et al. [27] found that different ionic PAMs regulate the properties of Portland cement through distinct mechanisms: the carboxyl groups in APAM markedly prolong hydration time and reduce early strength due to adsorption, while also increasing drying shrinkage and the proportion of air pores (1000 nm). In contrast, CPAM and NPAM promote the hydration heat release rate and have a minor impact on mechanical degradation. The three PAMs also differ in pore structure regulation: APAM refines the distribution, CPAM expands it, and NPAM has no significant effect. Consequently, these different ionic groups may exhibit varying electrostatic interactions (attraction or repulsion) with the ions in the MPC hydration system (e.g., Mg^2+^, K^+^, HPO_4_^2−^), resulting in differences in hydration rates, microstructures, mechanical and electrochemical performance of MPC. In particular, the mechanism by which the charge characteristics of different ionic PAMs influence the energy storage performance of MPC holds significant research value.

This study aims to systematically investigate the influence of different ionic PAMs and their dosages on the hydration process, microstructural evolution, and electrochemical energy storage characteristics of MPC. By integrating microscopic characterizations (XRD, FTIR, MIP) with electrochemical evaluations (CV, GCD, EIS), the internal mechanism of ionic group-regulated “structure-performance” is revealed. This work provides a theoretical foundation for the development of novel building energy storage materials with high energy density and structural stability.

## 2. Materials and Methods

### 2.1. Raw Materials

Dead-burned magnesia (MgO, CAS-1309-48-4, 98%, LR), potassium dihydrogen phosphate (KH_2_PO_4_, CAS-7778-77-0, 99.5%, LR), Cationic Polyacrylamide (CPAM, CAS-9003-05-8, Mw ≈ 1.2 × 10^7^), Anionic Polyacrylamide (APAM, CAS-9003-05-8, Mw ≈ 1.2 × 10^7^), and Non-Ionic Polyacrylamide (NPAM, CAS-9003-05-8, Mw ≈ 1.2 × 10^7^) were purchased from Shanghai Macklin Biochemical Co. Ltd., Shanghai, China. Borax (Na_2_B_4_O_7_·10H_2_O, CAS-1303-96-4, purity ≥ 99%), Potassium hydroxide (KOH, CAS-1310-58-3, 85%, GR), Hydrochloric acid (HCl, CAS-7647-01-0, 36~38%), Polyvinylidene fluoride (PVDF, CAS-24937-79-9, Mw ≈ 400,000), N-methyl-2-pyrrolidone (NMP, CAS-872-50-4, >99.5%), Acetylene black, and Anhydrous ethanol (CAS-64-17-5, ≥99.7%) were purchased from Sinopharm Chemical Reagent Co. Ltd., Shanghai, China. Activated carbon (AC) was obtained from Kuraray Co. Ltd., Osaka, Japan. Stainless steel plates with a thickness of 0.1 mm were purchased from Shandong Kerui Co. Ltd., Jinan, China. Nickel foam was supplied by Jiangsu Zhenyuhong New Material Co., Ltd., Suqian, China. The nickel foam has a continuous porous structure with an areal density of 355 ± 30 g m^−2^ and a thickness of 1 ± 0.1 mm. All chemical reagents were used without further purification.

### 2.2. Preparation of the Activated Carbon (AC) Electrodes

First, the nickel foam of 1 cm × 2 cm was activated by cleaning with dilute hydrochloric acid (HCl, 36~38%), followed by repeated rinsing with ethanol and deionized water, and then dried at 60 °C. Activated carbon (AC), polyvinylidene fluoride (PVDF), and carbon black were uniformly mixed at a mass ratio of 8:1:1. An appropriate amount of N-methyl-2-pyrrolidone (NMP) was added dropwise to the mixture, which was continuously ground until a homogeneous ink-like slurry was obtained. Subsequently, the slurry was coated onto the activated nickel foam. The AC-coated nickel foam was dried in a vacuum oven at 60 °C. Finally, the AC-coated nickel foam was wrapped in weighing paper and pressed under a pressure of 10 MPa.

### 2.3. Synthesis of the Polyacrylamide-Magnesium Phosphate Cement (PAM-MPC) Composites

Dead-burned magnesia (MgO), potassium dihydrogen phosphate (KH_2_PO_4_), borax (Na_2_B_4_O_7_·10H_2_O), and PAM were homogeneously mixed according to the proportions as shown in Table 1 and stirred for 3 min. The resulting homogeneous paste was cast into two types of cubic molds (1 cm^3^ and 64 cm^3^) and subsequently cured for 28 days under laboratory environmental conditions (approximately 20 °C and 52% relative humidity). The water-to-cement ratio (*w*/*c*) was fixed at 0.6. Cubic specimens of 1 cm^3^ were utilized for ionic conductivity measurements, whereas larger specimens of 64 cm^3^ were employed for compressive strength testing.

### 2.4. Fabrication of the Cementitious Structural Supercapacitors (CSSCs)

**Figure 1:**
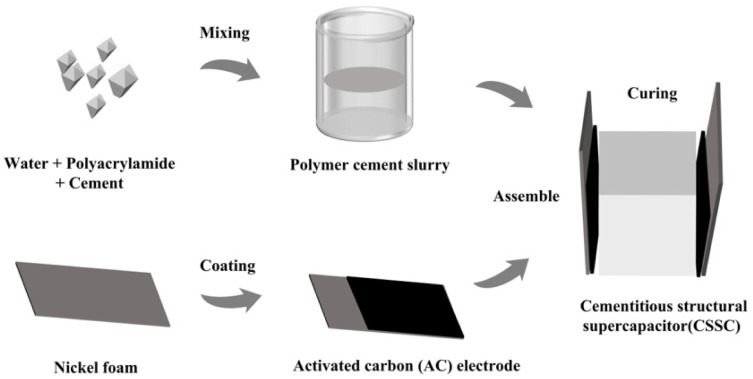
XRD patterns of MPC electrolytes modified with 0.5% and 2.0% dosages of NPAM, CPAM, and APAM.

First, the homogeneous PAM-MPC slurry was cast into a mold (10 mm × 10 mm × 5 mm) with electrodes embedded on both sides to form a sandwich-style assembly, thereby obtaining a symmetric cement-based supercapacitor, as shown in Figure 1. The assembled supercapacitor was cured in ambient air, then demolded, and further cured in air till the specified age for testing.

### 2.5. Microstructural Characterization and Performance Testing

A scanning electron microscope (SU8600, Hitachi, Tokyo, Japan) integrated with an Oxford Instruments Xplore spectrometer was utilized. In preparation for SEM testing, fragments were taken from the inner part of the freshly fractured specimens. All samples were then analyzed at an accelerating voltage of 5kV and 20 kV in the secondary electron (SE) mode.

The pore structure of the samples was analyzed using an automated mercury intrusion porosimeter (MIP, Micromeritics, Norcross, GA, USA. The compressive strength of the specimens was measured using a universal testing machine (JES-300 Concrete Compression Tester, Manufacturer unknown, Shanghai, China). X-ray diffraction (XRD) patterns were recorded using a Bruker diffractometer (Bruker, Karlsruhe, Germany) with Cu Kα radiation (λ = 0.154 nm, 35 kV, 25 mA) in the 2θ range of 5–85°.

Fourier transform infrared (FTIR) measurements were conducted on a Thermo Scientific Nicolet Summit X spectrometer (Thermo Fisher Scientific, Waltham, MA, USA) with a scanning resolution of 4 cm^−1^, covering a spectral range from 400 cm^−1^ to 4000 cm^−1^. Approximately 1 mg of the powdered sample was mixed with 100 mg of KBr to form pellets through compression, which were then subjected to the tests.

The setting time was measured in accordance with the Chinese standard GB/T 1346–2011 utilizing the Vicat apparatus. Both the initial setting time (IST) and final setting time (FST) of the MPC were tested. For the experimental groups, the testing intervals for their setting time were 5 s and 30 s, respectively. The initial setting time was determined when the needle penetrated to 4 mm ± 1 mm above the bottom plate, while the final setting time was determined when the needle penetration into the mixture was less than 0.5 mm. The density of MPC is the ratio of the mass to the volume of the samples.

Electrochemical measurements, including cyclic voltammetry (CV), galvanostatic charge–discharge (GCD), and electrochemical impedance spectroscopy (EIS), were performed on a CHI660E electrochemical workstation (Shanghai Chenhua, Shanghai, China).

For EIS testing, the electrolyte was sandwiched between two stainless steel electrodes. The measurements were conducted in the frequency range of 0.01 Hz–100 kHz with an applied voltage amplitude of 5 mV. The bulk resistance of the electrolyte was determined by the intercept of the Nyquist plot on the real axis, and the ionic conductivity of the sample was calculated using the following Equation (1):

(1) $$\sigma =\frac{L}{R\cdot A}$$

where *σ* (mS cm^−1^), *L* (cm) and *A* (cm^2^) represent the ionic conductivity, thickness and working area of the sample, respectively. *R* (Ω) is the bulk resistance.

In two-electrode characterization of the symmetric CSSC, *q* (mC) is calculated from GCD curves using Equation (2):

(2) q=I⋅t

where *q* (mC) is the total amount of charge stored, *I* (mA) is the discharge current, and *t* (s) is the discharge time. The capacitance *C* (mF) of devices is calculated using Equation (3):

(3) $$C=\frac{q}{V}$$

where *V* (V) is the potential change during discharge.

The specific capacitance *C_sp_,* such as areal capacitance *C_α_* (mF cm^−2^), are calculated using Equation (4):

(4) $$C_{a}=\frac{C}{S}$$

where *C* (mF) is the capacitance, and *S* (cm^2^) is the active electrode area.

The areal energy density *E_a_* (mWh cm^−2^) of a symmetric CSSC is calculated from GCD curves using Equation (5):

(5) $$E_{a}=\frac{C_{a}\cdot V^{2}}{2\cdot 3600}$$

where *C_a_* (mF cm^−2^) is the areal capacitance obtained from Equation (4), and V (V) is the potential change during the discharge.

## 3. Results and Discussion

### 3.1. Microstructural Characterization

#### 3.1.1. XRD Analysis

**Figure 2:**
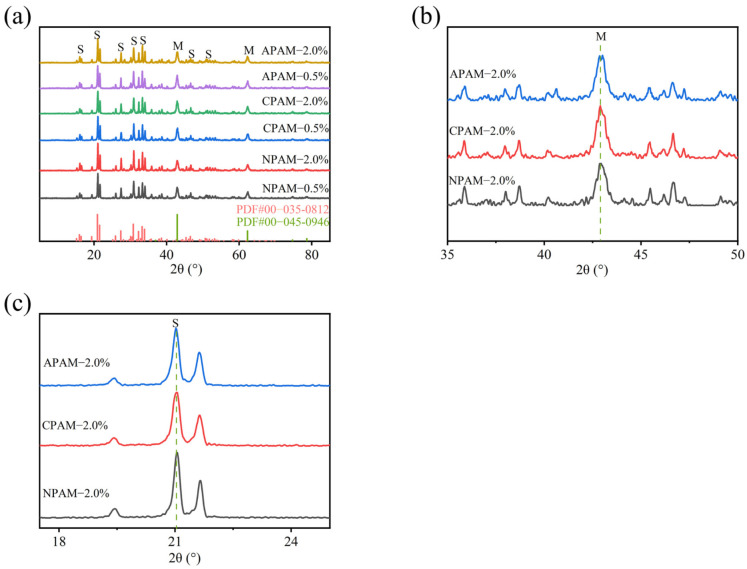
(**a**) XRD patterns of MPC electrolytes modified with 0.5% and 2.0% dosages of NPAM, CPAM, and APAM; (**b**,**c**) show the same patterns, focusing on the 2θ ranges of 35–50° and 17–25°, respectively.

To elucidate the hydration products, XRD analysis was conducted on PAM-modified MPC to characterize the solid-phase composition after 28 days. As shown in Figure 2a, the main characteristic peaks of PAM-modified MPC were identified as magnesium oxide (MgO) and K-struvite (KMgPO_4_·6H_2_O). The peaks located at 21°, 27.4°, 30.9° and 33.3° correspond to K-struvite, while the diffraction peaks at 42.9° and 62.3° are assigned to MgO. No new crystalline phases were detected, indicating that the incorporation of PAM is primarily via physical mixing or surface chemical modification [29,30,31,32,33]. With increasing dosage, the differences in diffraction peak intensities among MPC modified with different ionic PAMs became increasingly significant. At a low dosage (0.5%), there were no distinct differences in the diffraction peaks among the three groups. However, at a high dosage (2.0%) as shown in Figure 2b,c, the intensity of MgO characteristic peaks followed the order: NPAM-2.0% < APAM-2.0% < CPAM-2.0%. Conversely, the intensity of K-struvite characteristic peaks followed the order: NPAM-2.0% > APAM-2.0% > CPAM-2.0%. In addition, we found that the peak width of magnesium oxide (MgO) showed no obvious differences among all groups with different ionic PAM types and dosages (0.5% and 2.0%), indicating that the crystal grain size and crystallinity of unreacted MgO were not significantly affected by the introduction of PAMs. In contrast, the peak widths of the characteristic peaks of potassium struvite (K-struvite), the main hydration product of MPC, exhibited a distinct trend with the order of CPAM-2.0 > APAM-2.0% > NPAM-2.0%. This phenomenon is consistent with the trend observed in the characteristic peak intensities. These results indicate that upon reaching a certain dosage, the functional groups of different ionic PAMs exert significantly different effects on the formation of K-struvite, following the trend: NPAM < APAM < CPAM. This can be attributed to the fact that the functional groups of APAM are negatively charged, while those of CPAM are positively charged. Both can generate electrostatic interactions with charged ions in the cement paste, thereby significantly inhibiting the growth of the hydration product, K-struvite. In contrast, the functional groups of NPAM primarily interact via hydrogen bonding, which has a relatively minor impact on the formation of MPC hydration products.

#### 3.1.2. FTIR Analysis

**Figure 3:**
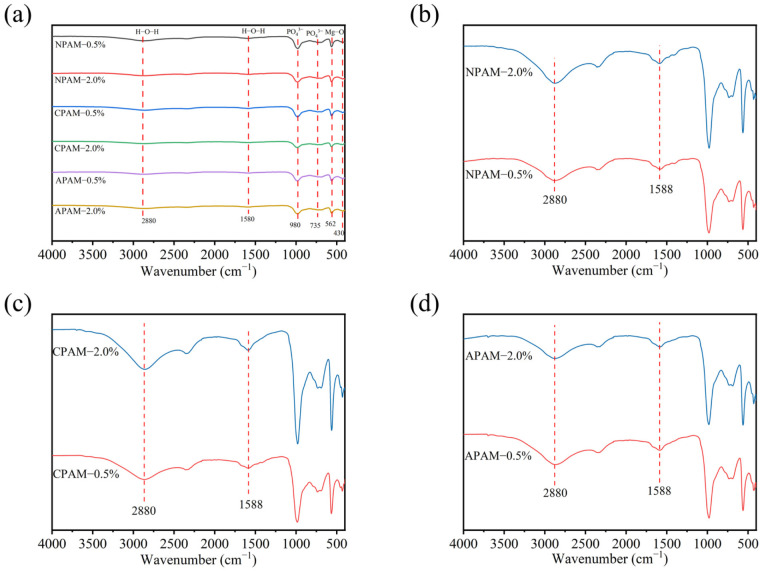
FTIR spectra of MPC electrolytes modified-with 0.5% and 2.0% dosages of: (**a**) NPAM, CPAM and APAM, (**b**) NPAM. (**c**) CPAM, (**d**) APAM.

XRD analysis confirmed the presence of substantial amounts of K-struvite and unreacted MgO. As shown in Figure 3, FTIR also revealed characteristic absorption peaks for these phases. The peaks located at 430 cm^−1^ and 562 cm^−1^ are attributed to Mg-O bonds. The most prominent characteristic peak of the K-struvite at 980 cm^−1^ corresponds to the antisymmetric stretching vibration of PO_4_^3−^, while the peak at 735 cm^−1^ corresponds to the bending vibration of PO_4_^3−^ [18,34]. As shown in Figure 3b–d, when the polymer dosage increases from 0.5% to 2.0%, the peak at 1588 cm^−1^ gradually broadens and a new peak tendency appears in the region of 1600–1700 cm^−1^. This evolution can be attributed to the superposition of the H-O-H bending vibration of crystal water and the amide I band of PAM in this wavenumber range [34,35,36]. The peak located at 2880 cm^−1^, as observed in Figure 3b–d, appears as a broad band rather than a sharp peak. This indicates that the N–H stretching vibration from the amide groups of PAM is masked by the downshifted O–H stretching vibration of water molecules in struvite-K with strong hydrogen-bonding interactions [34,36,37]. All peak information can be found in Supplementary Material Table S1. Compared to APAM-0.5%, the antisymmetric stretching vibration peak of PO_4_^3−^ in APAM-2.0% exhibited a red-shift. This is attributed to the increased dosage of APAM, which enhances the electrostatic interaction between carboxyl groups and ions in the cement, interfering with ionic bond formation and causing the red-shift of the PO_4_^3−^ peak [36,37]. The PO_4_^3−^ peak position for CPAM-2.0% showed a blue-shift compared to CPAM-0.5%, suggesting that the electrostatic interaction between cationic groups and ions in the cement intensifies with increasing dosage, affecting the crystallization of K-struvite [15,30]. The PO_4_^3−^ peak positions for NPAM-0.5% and NPAM-2.0% showed no significant changes, indicating that NPAM mainly acts through hydrogen bonding and has a minimal effect on MPC hydration products. Thus, the FTIR results further confirm the differential effects of functional groups from different ionic PAMs on K-struvite formation.

#### 3.1.3. SEM and EDS Analysis

**Figure 4:**
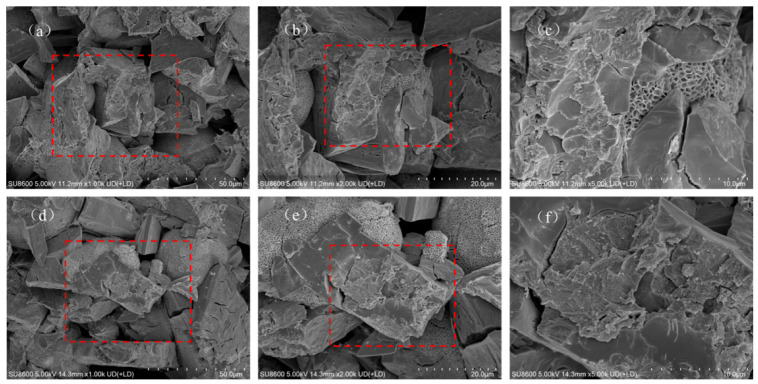
SEM images of (**a**–**c**) NPAM-0.5% and (**d**–**f**) NPAM-2.0%, red square marks the magnified region.

**Figure 5:**
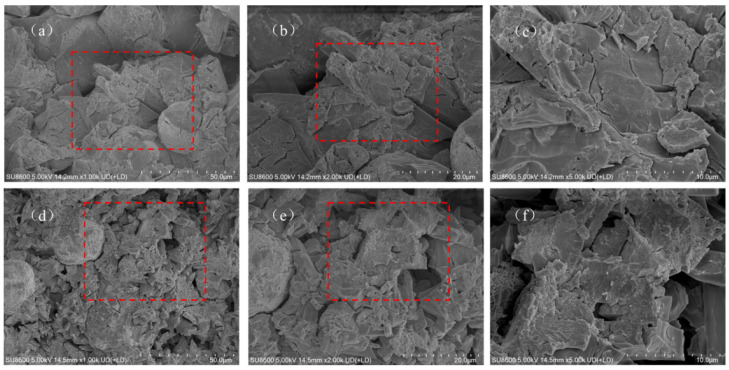
SEM images of (**a**–**c**) CPAM-0.5% and (**d**–**f**) CPAM-2.0%, red square marks the magnified region.

**Figure 6:**
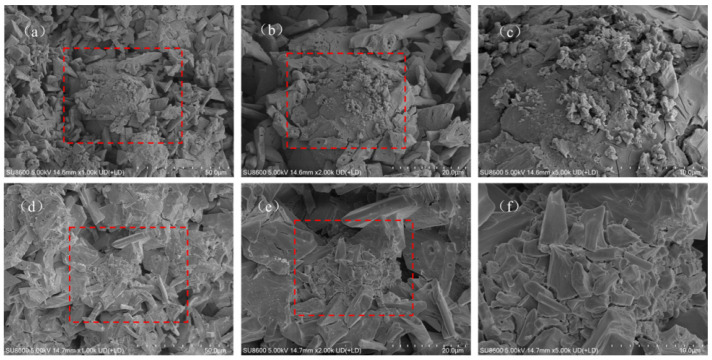
SEM images of (**a**–**c**) APAM-0.5% and (**d**–**f**) APAM-2.0%, red square marks the magnified region.

SEM images of MPC modified by different ionic PAMs revealed the evolution patterns of the cement micromorphology. The effects were closely related to the dosage. The SEM images of NPAM-0.5% (Figure 4a–c) show that it maintain intact columnar or plate-like characteristics. With the dosage increased to NPAM-2.0% (Figure 4d–f), the edges of the K-struvite crystals became smooth, and the polymer film physically encapsulated the hydration products. Only a minimal quantity of honeycomb-like polymer was observed in the images of NPAM-modified MPC. The SEM images of CPAM-0.5% (Figure 5a–c) show more ellipsoidal morphologies. As the dosage increased to CPAM-2.0% (Figure 5d–f), a small amount of honeycomb-like polymer and ellipsoidal morphologies increased. The SEM images of APAM-0.5% (Figure 6a–c) show obvious honeycomb-like polymer and a small amount of ellipsoidal morphologies. With the dosage increased to APAM-2.0% (Figure 6d–f), the honeycomb-like polymer and the ellipsoidal morphologies increased.

**Figure 7:**
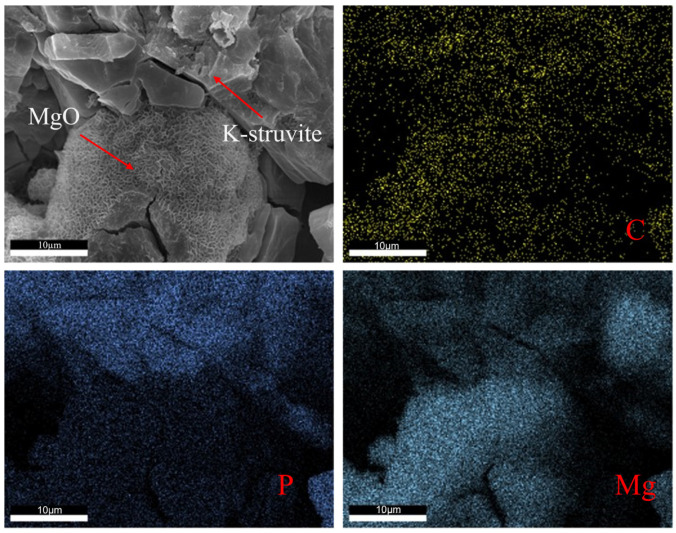
SEM-EDS images of APAM-2.0% showing the distribution of C, P, and Mg.

It is noteworthy that honeycomb-like polymer was observed in the SEM images of all samples (proportion of honeycomb-like polymer: NPAM < CPAM < APAM), with APAM-2.0% exhibiting the highest proportion. The combined SEM-EDS analysis of APAM-2.0% (Figure 7) shows a homogeneous distribution of carbon (C). High-intensity phosphorus (P) signals were concentrated in K-struvite crystals, contrasting with the polymer-wrapped regions where magnesium (Mg) signals were dominant and P signals were diminished. This is attributed to the anionic side chains of APAM chemically cross-linking with Mg^2+^ and K^+^ to form a network structure that adsorbs onto the MgO surface, which is unfavorable for K-struvite crystal formation.

**Figure 8:**
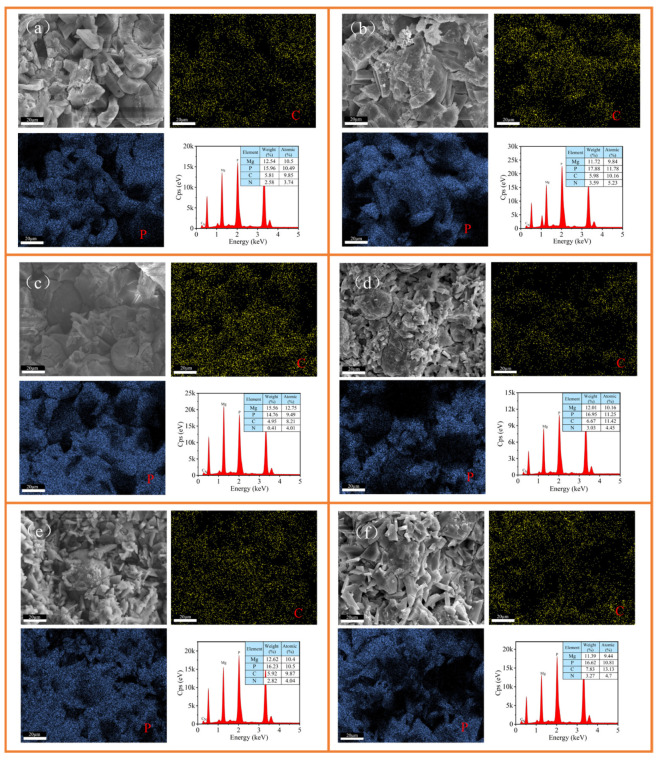
SEM and EDS images of (**a**) NPAM-0.5%, (**b**) NPAM-2.0%, (**c**) CPAM-0.5%, (**d**) CPAM-2.0%, (**e**) APAM-0.5%, and (**f**) APAM-2.0%.

The EDS elemental distribution results are combined to further analyze the intrinsic mechanism. At a dosage of 0.5% (Figure 8a,c,e), the C and P signals in NPAM-0.5% were uniformly distributed, indicating no significant impact on the formation of K-struvite. However, in CPAM-0.5% (Figure 8c), a small ellipsoidal region exists where the P signal distribution was uneven. This indicated that K-struvite formation was hindered in this area, leaving a small amount of unreacted MgO. This is attributed to the cationic side chains of CPAM generating electrostatic interactions with anions, thereby hindering the participation of HPO_4_^2−^ in the hydration reaction. Similarly, the P signal distribution in the ellipsoidal morphology regions of APAM-0.5% (Figure 8e) was uneven. This originated from the electrostatic interactions between the anionic side chains of APAM and cations, which hindered the participation of Mg^2+^ and K^+^ in the hydration reaction. When the dosage reached 2.0%, the C and P signals in NPAM-2.0% (Figure 8b) were basically consistent, indicating that the polymer and K-struvite crystals were uniformly distributed. This is attributed to the fact that the functional groups of NPAM primarily interact via hydrogen bonding, which has almost no effect on the formation of K-struvite. In the images of CPAM-2.0% and APAM-2.0% (Figure 8d,f), the shrinkage of the P signal area indicates a reduction in the amount of K-struvite crystals. This is primarily attributed to the enhanced electrostatic interaction induced by the polymer addition, which further restricted the formation of K-struvite.

#### 3.1.4. MIP Analysis

Previous studies have indicated that the cement pores can be classified into gel pores (<10 nm), capillary pores (10–1000 nm), and air voids (>1000 nm). The pore structure of cement electrolytes is pivotal to their electrochemical energy storage performance and mechanical properties, as it influences the electrochemical reaction rates, ion transport, mechanical strength, and thermal stability. Therefore, the MIP was conducted to quantitatively characterize the pore structure and distribution of PAM-modified cement specimens (28 days).

**Figure 9:**
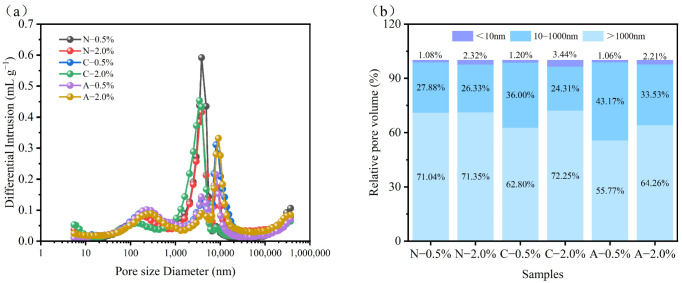
(**a**) Pore size distributions and (**b**) proportions of pores with sizes of 0–10 nm, 10–1000 nm, and >1000 nm for MPC electrolytes modified with 0.5% and 2.0% dosages of NPAM, CPAM, and APAM.

The MIP results (Figure 9a,b) reveal significant differences in the pore size distribution of MPC modified with different ionic PAMs. With increasing dosage, the proportions of micropores (<10 nm) and macropores (>1000 nm) in the samples showed an increasing trend, whereas the proportion of mesopores (10–1000 nm) decreased. Specifically, the extent of micropore increase followed the order CPAM > NPAM > APAM, while the order for macropore increase was CPAM > APAM > NPAM. Conversely, the reduction in mesopores was most pronounced in CPAM, followed by APAM and NPAM (CPAM > APAM > NPAM). These results indicate that, with increasing polymer dosage, the regulation effect on pore size distribution varies significantly among the groups. Specifically, NPAM exhibited the weakest regulatory effect, inducing only a slight increase in micropore proportion. This was followed by APAM, which increased both micropore and macropore proportions. However, the macropore fraction in APAM-2.0% remained the lowest among the three groups (64.26%). In contrast, CPAM demonstrated the strongest regulation capability. The CPAM-2.0% sample displayed the most prominent pore size regulation characteristics, featuring the highest proportions of both micropores (3.44%) and macropores (72.25%). Based on the MIP analysis, pore refinement (increase in pores < 10 nm) contributes to enhanced compressive strength but leads to increased pore tortuosity, thereby hindering ion diffusion and reducing ionic conductivity. Conversely, an increase in macropores (>1000 nm) facilitates ion diffusion and boosts conductivity, but it significantly reduces the compressive strength of the cement electrolytes [38,39,40].

### 3.2. Performance Testing

#### 3.2.1. Compressive Strength

**Figure 10:**
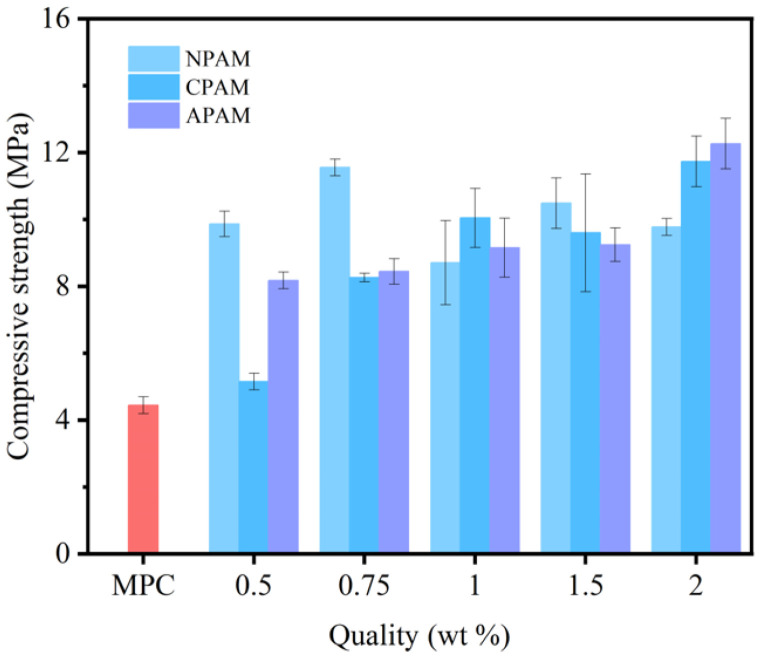
The compressive strength of MPC composites at 28 d.

The compressive strength of the three PAM-modified MPCs at 28 days is presented in Figure 10. Compared to the control group (MPC without PAM, 4.4 MPa), the compressive strengths of all three PAM-modified MPCs were improved. As the dosage of anionic polyacrylamide (APAM) increased, the APAM group demonstrated a consistent rise in strength, reaching a peak of 12.3 MPa (180% improvement) for the APAM-2.0% concentration. Similarly, the CPAM group increased to 11.7 MPa (165% improvement) for CPAM-2.0%. In contrast, the NPAM group fluctuated, reaching a maximum of 11.6 MPa (164% improvement) at 0.75% dosage. Correlating with the microstructural characterization results, the strength trends of APAM- and CPAM-modified MPC are attributed to the synergy between the polymer’s excellent bonding properties and the optimized pore size distribution. This synergy effectively compensates for the strength loss caused by macropores, thereby promoting strength development. The strength improvement of NPAM-modified MPC is attributed to the excellent bonding performance of the polymer, which effectively bonds cement particles and fills micro-cracks to compensate for the strength loss induced by macropores. However, when the NPAM dosage exceeded the optimal threshold, the polymer enveloped the raw materials and severely hindered the hydration process, which resulted in a decrease in strength.

#### 3.2.2. Setting Properties of PAM-Modified MPC

**Figure 11:**
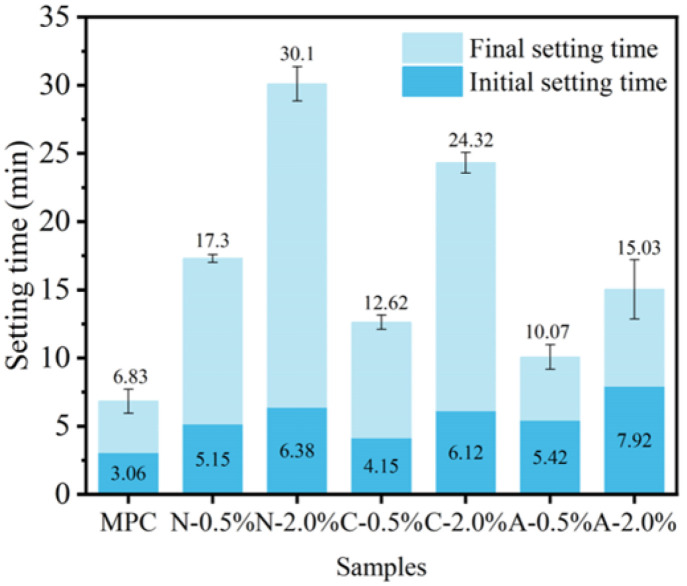
Setting times of MPC with different ionic PAMs at 0.5% and 2.0% dosages.

As illustrated in Figure 11, PAMs significantly retarded the setting and hardening of MPC. The retarding effect of all three PAM types was enhanced with increasing dosage. NPAM exhibited the strongest retardation: NPAM-0.5% exhibited the longest final setting time of 17 min at a dosage of 0.5%, whereas NPAM-2.0% extended to 30 min, surpassing the control by a factor of four. CPAM followed by an intermediate effect, characterized by rapid initial setting but delayed final setting: it recorded the shortest initial setting times (4 min at 0.5%, 6 min at 2.0%). Conversely, APAM showed the weakest retardation, featuring delayed initial setting but rapid final setting; it yielded the longest initial setting times (5 min 30 s at 0.5%, 8 min at 2.0%) but the shortest final setting times (10 min at 0.5%, 15 min at 2.0%). This can be attributed to their distinct functional groups. Specifically, the carboxyl groups (-COOH) in APAM interact electrostatically with K^+^ and Mg^2+^, thereby hindering their participation in hydration. Similarly, the quaternary ammonium groups (-N^+^(CH_3_)_3_) present in CPAM interact with HPO_4_^2−^, thereby restricting its reaction [28,41]. In contrast, NPAM achieves retardation primarily through the physical wrapping of the raw materials, which inhibits their dissolution.

#### 3.2.3. Electrochemical Properties

**Figure 12:**
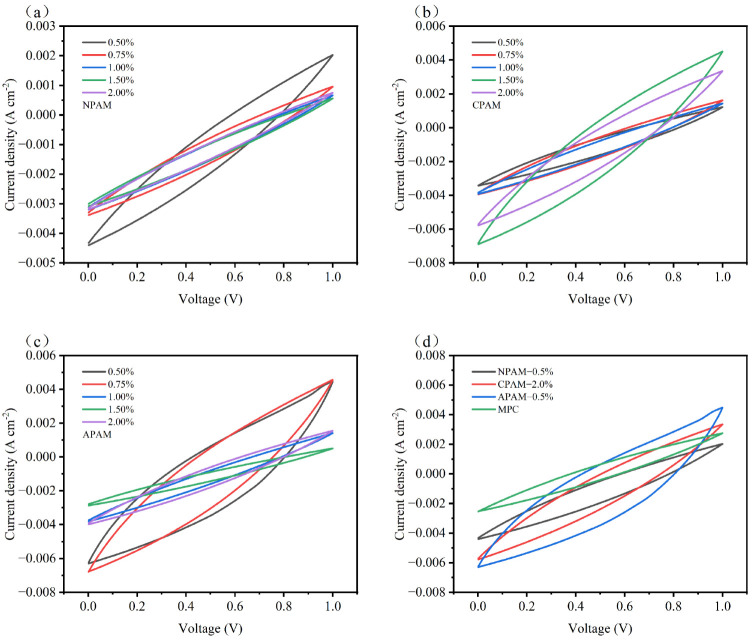
CV curves of magnesium phosphate cement (MPC) supercapacitors at a scan rate of 10 mV s^−1^ with: (**a**) NPAM, (**b**) CPAM, and (**c**) APAM at dosages ranging from 0.5% to 2.0%. (**d**) Comparative CV curves of supercapacitors with NPAM-0.5%, CPAM-2.0%, and APAM-0.5%.

CSSCs are assembled from activated carbon electrodes and the MPC electrolytes modified by different ionic PAMs. These supercapacitors are designated as NPAM-CSSC, CPAM-CSSC, and APAM-CSSC according to their respective polymer types. The electrochemical performance of the CSSCs was rigorously assessed using standard methods such as cyclic voltammetry (CV), galvanostatic charge–discharge (GCD), and electrochemical impedance spectroscopy (EIS). The quasi-rectangular CV shapes at a scan rate of 10 mV s^−1^ (Figure 12a–d) indicate that the energy storage mechanism of all CSSCs is dominated by electric double-layer capacitance (EDLC) [42,43,44]. Figure 12a,c show that for NPAM-CSSC and APAM-CSSC, the integrated area of the CV curves decreased with increasing dosage, with NPAM-0.5% and APAM-0.5% exhibiting the largest integrated areas. Conversely, Figure 12b demonstrates that the integrated area of the CV curves for CPAM-CSSC increased with increasing dosage, with CPAM-2.0% showing the largest area. The CV results suggest that NPAM-0.5%, CPAM-2.0%, and APAM-0.5% possess the optimal charge storage capability, as a larger integrated area in the CV curve signifies superior charge storage capacity. Figure 12d reveals that the integrated areas follow the order: APAM-0.5% > CPAM-2.0% > NPAM-0.5% > MPC, indicating that the charge storage capability of these CSSCs follows the same order.

**Figure 13:**
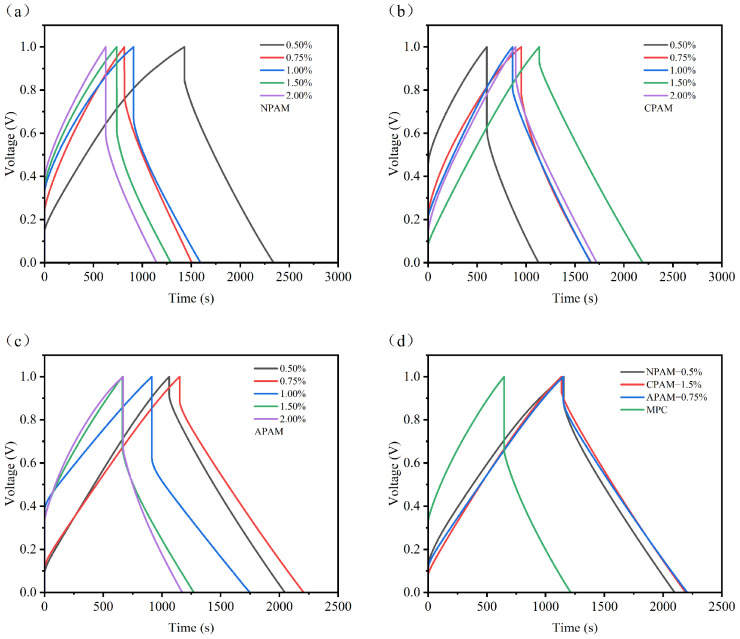
GCD curves of MPC supercapacitors at a current density of 1 mA cm^−2^ with: (**a**) NPAM, (**b**) CPAM, and (**c**) APAM at dosages ranging from 0.5% to 2.0%. (**d**) Comparative GCD curves of supercapacitors with NPAM-0.5%, CPAM-2.0%, and APAM-0.5%.

At a current density of 1 mA cm^−2^, Figure 13a shows that the trend of the GCD curves for NPAM-CSSC is consistent with that of the CV curves, with NPAM-0.5% exhibiting the longest discharge time. Figure 13b,c indicate that for CPAM-CSSC and APAM-CSSC, the discharge time initially increased and then decreased with increasing dosage, with CPAM-1.5% and APAM-0.75% showing the longest discharge times, respectively. Figure 13d demonstrates that the discharge times and the symmetry of the triangular shape for NPAM-0.5%, CPAM-1.5%, and APAM-0.75% are all superior to those of the pristine MPC. This suggests that these modified samples possess superior charge storage capability, better Coulombic efficiency, and enhanced electrochemical reversibility.

**Figure 14:**
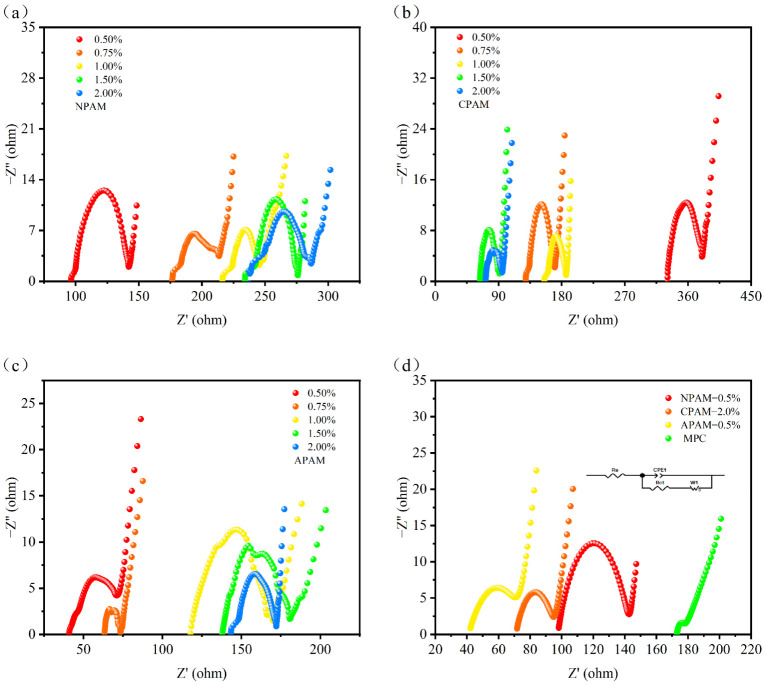
Nyquist plots of electrochemical impedance spectroscopy (EIS) for magnesium phosphate cement (MPC) supercapacitors in the frequency range of 0.01 Hz–100 kHz with: (**a**) NPAM, (**b**) CPAM, and (**c**) APAM at dosages of 0.5–2.0%. (**d**) Comparative Nyquist plots for supercapacitors with NPAM-0.5%, CPAM-2.0%, and APAM-0.5%.

Furthermore, EIS measurements were conducted in the frequency range of 0.01 Hz to 100 kHz to reveal the charge transfer and ion migration behaviors within the CSSCs. Rs is defined as the equivalent series resistance, which includes the internal resistance, while Rct corresponds to the charge transfer resistance (or diffusion resistance) at the electrode/electrolyte interface. Figure 14a–c show that with increasing dosage, the Rs of APAM-CSSCs and NPAM-CSSCs increased, suggesting better capacitive performance at low dosages (0.5% and 0.75%); the Rs of CPAM-CSSCs decreased, suggesting high dosages (1.5% and 2.0%) are superior. Figure 14d reveals that the Rs values for MPC, NPAM-0.5%, CPAM-2.0%, and APAM-0.5% CSSCs were 175 Ω, 97.6 Ω, 71 Ω, and 41.1 Ω, respectively. Among them, the APAM-0.5% CSSC exhibited the lowest Rs value, suggesting it possesses the best capacitive characteristics. The Rct values for APAM-0.5% and CPAM-2.0% were 34 Ω and 25 Ω, respectively, which were lower than that of NPAM-0.5% (45 Ω), indicating lower diffusion resistance for the former two. We observed that while the MPC CSSC had the highest Rs, it possessed the lowest Rct, suggesting that at certain dosages, PAM facilitates the enhancement of the capacitance of MPC but may exert an adverse effect on interfacial diffusion.

**Figure 15:**
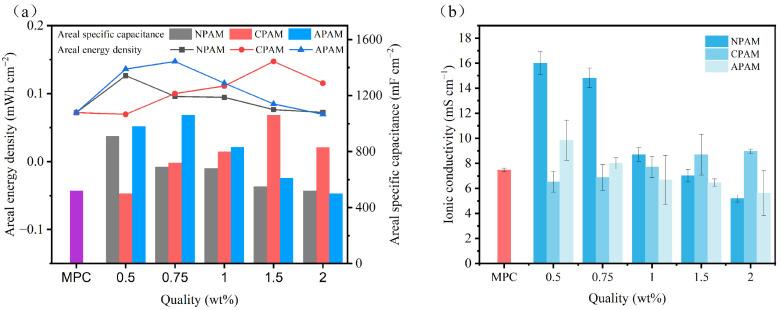
(**a**) Areal capacitance and areal energy density curves of CSSCs with NPAM, CPAM, and APAM at dosages of 0.5–2.0% at a current density of 1 mA cm^−2^, and (**b**) ionic conductivity of the electrolytes.

Quantitative analysis of the areal specific capacitance and energy density for NPAM-CSSC, CPAM-CSSC, and APAM-CSSC is shown in Figure 15a. We found that the areal capacitance and energy density of NPAM-CSSC and APAM-CSSC decreased with increasing dosage, with NPAM-0.5% reaching maxima of 910 mF cm^−2^ and 0.126 mWh cm^−2^, and APAM-0.75% reaching 1060 mF cm^−2^ and 0.147 mWh cm^−2^. In contrast, CPAM-CSSC showed an initial increase followed by a decrease with increasing dosage, peaking at CPAM-1.5% with 1060 mF cm^−2^ and 0.147 mWh cm^−2^. Compared to the areal capacitance and energy density of the control group (520 mF cm^−2^ and 0.072 mWh cm^−2^), it was found that at appropriate dosages, all three PAM-modified MPC supercapacitors exhibited improvements in both metrics (magnitude: CPAM > APAM > NPAM), with a maximum increase of 104%. This further confirms that different ionic PAMs have distinct effects on the electrochemical performance of CSSCs, and at specific dosages, they are beneficial for enhancing the charge storage capability of MPC capacitors.

Figure 15b presents the ionic conductivity of NPAM, CPAM, and APAM at dosages of 0.5–2.0%. Compared with the control group, the ionic conductivity of MPC modified with all three ionic PAMs increased at appropriate dosages (order: NPAM > APAM > CPAM). The ionic conductivity of the NPAM and APAM groups showed a decreasing trend with increasing dosage, whereas the CPAM group showed the opposite trend. This difference is attributed to the impact of the microstructure regulated by different PAMs on the ion transport. It is noteworthy that the ionic conductivity of NPAM-0.5% was the highest, reaching 16 mS cm^−1^, which is twice that of the control group. Nevertheless, the CSSC based on NPAM-0.5% failed to attain the optimal electrochemical performance. This suggests that the electrochemical energy storage properties of CSSCs are influenced by a range of factors, such as the choice of electrolyte, the design and material of the electrode, and the characteristics of the electrode-electrolyte interface, necessitating a holistic approach to their analysis.

**Figure 16:**
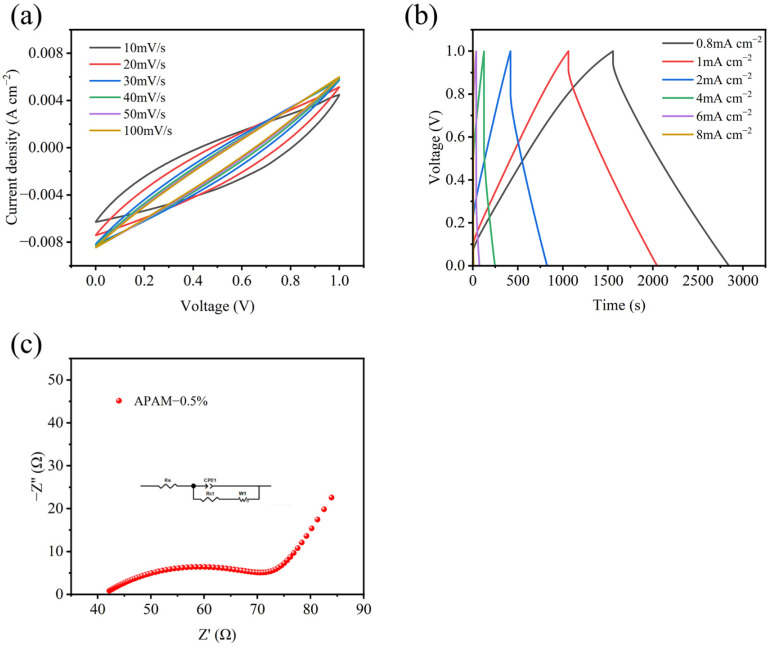
Electrochemical performance of the APAM-0.5%CSSC: (**a**) CV curves at different scan rates of 10–100 mV s^−1^, (**b**) GCD curves at different current densities of 0.8–8 mA cm^−2^, (**c**) EIS curve in the frequency range of 0.01 Hz–100 kHz.

Considering the electrochemical performance, mechanical properties, and cost-effectiveness, APAM-CSSC at 0.5% dosage is the optimal choice. It has a low polymer dosage while simultaneously possessing favorable electrochemical and mechanical properties. Therefore, we evaluated the CV (Figure 16a) at different scan rates (10–100 mV s^−1^) and GCD (Figure 16b) at different current densities (0.8–8 mA cm^−2^) for the APAM-CSSC. The quasi-rectangular CV shapes at different scan rates indicate that the energy storage mechanism is dominated by electric double-layer capacitance (EDLC). The GCD curves all exhibited approximately linear and highly symmetrical characteristics, demonstrating its excellent charge storage capability. At current densities of 4, 2, 1, and 0.8 mA cm^−2^, its areal capacitances were 490, 810, 980 and 1030 mF cm^−2^, respectively, indicating good rate capability. Furthermore, the fitted EIS curve (Figure 16c) was plotted within the frequency range of 0.01 Hz–100 kHz. Its Rs and Rct values were only 41.1 Ω and 34 Ω, respectively, indicating that it possesses excellent capacitive characteristics and low diffusion resistance.

#### 3.2.4. Influencing Mechanism of Different Ionic Types of PAMs on the Performance of CSSCs

Through microstructural characterization (MIP, SEM, FTIR, etc.) and macro-performance testing (ionic conductivity, compressive strength, electrochemical energy storage characteristics, etc.), the relationships between the mechanical properties, ionic conductivity, and electrochemical performance of MPC samples modified with different ionic PAMs and their microstructures were systematically investigated.

Compared to the control group, the NPAM-modified MPC samples exhibited a significantly prolonged hydration rate. At appropriate dosages, both their mechanical properties and electrochemical performance were improved. For instance, NPAM-0.5% exhibited excellent synergistic effects between ionic conductivity and mechanical properties. It possessed a high proportion of macropores (>1000 nm), and the ion transport channels constructed by these macropores significantly reduced ion migration resistance, endowing it with superior ionic conductivity. Meanwhile, the polymer effectively compensated for the strength reduction caused by the macroporous structure by means of bonding effects and filling micro-cracks. However, exceeding the dosage threshold limits compressive strength development, as excessive polymer wraps the raw materials and disrupts crystal skeleton formation. Furthermore, the decrease in ionic conductivity can be attributed to the swelling of excessive polymer during testing, which blocks ion diffusion channels.

For CPAM-modified MPC, compared to the control group, both mechanical properties and electrochemical performance were enhanced at appropriate dosages, showing an increasing trend with higher dosages. This can be attributed to CPAM having the strongest influence on the pore structure; increasing the dosage significantly raised the proportion of macropores (>1000 nm), facilitating the improvement of ionic conductivity. However, it also increased the proportion of micropores (<10 nm), which restricts further enhancement of ionic conductivity. Theoretically, an increase in macropores in MPC is detrimental to strength development. Nevertheless, pore size refinement and the polymer binding the dispersed hydration product particles balanced the strength loss caused by the macroporous structure. For example, CPAM-2.0% exhibited excellent synergistic effects of ionic conductivity and mechanical properties.

The incorporation of APAM into MPC has been shown to enhance both mechanical properties and electrochemical performance at optimal concentrations, as evidenced by similar material modifications detailed in the previous study [40]. However, increasing the dosage created a trade-off between compressive strength and ionic conductivity. This can be attributed to three factors: (1) like CPAM, APAM compensates for the strength loss from macropores through pore refinement and excellent polymer bonding; (2) although APAM increased the proportion of pores > 1000 nm (beneficial for ions), the total macropore fraction remained the lowest, limiting conductivity; (3) during testing, excessive polymer swelled and blocked ion diffusion channels, reducing conductivity. Therefore, APAM-2.0% showed excellent mechanical properties, whereas APAM-0.5% exhibited superior electrochemical performance.

Finally, the comprehensive energy storage performance of supercapacitors depends on the synergistic interaction of the electrode, electrolyte, and electrode/electrolyte interface, rather than being determined solely by the electrolyte factor. For example, although the ionic conductivity of APAM-0.5% was lower than that of NPAM-0.5%, the charge transfer resistance (Rct) at the electrode/electrolyte interface for APAM-0.5% was significantly lower than that of NPAM-0.5% (34 Ω vs. 45 Ω), indicating better interfacial compatibility. Furthermore, CV tests confirmed that APAM-0.5% possessed a more symmetrical rectangular curve and a larger integrated area, reflecting a stronger charge storage capability.

## 4. Conclusions

This study systematically investigated the influence of three different ionic PAMs on the hydration, mechanical properties, and energy storage characteristics of modified MPC. The following conclusions were drawn:

(1) All three ionic PAMs reduced the hydration rate and retarded the hydration process of MPC. Among them, NPAM exhibited the strongest retarding effect, followed by CPAM and APAM sequentially. These effects became more significant with increasing PAM dosage.

(2) The incorporation of NPAM did not notably alter the hydration product, K-struvite, within MPC. Conversely, both APAM and CPAM were observed to have a significant impact on the development of K-struvite in MPC.

(3) All three ionic PAMs optimized the pore size distribution. NPAM did not significantly increase the proportion of macropores (air voids) in MPC. Conversely, both APAM and CPAM significantly increased the proportion of macropores, with CPAM exhibiting the most significant effect.

(4) At dosages ranging from 0.5% to 2.0%, the compressive strength of MPC was improved by the incorporation of all three types of ionic PAMs. With increasing dosages, both the APAM and CPAM groups demonstrated a monotonically increasing trend in compressive strength, while the NPAM group experienced an increase before generally declining.

(5) At appropriate dosages, the electrochemical performance of supercapacitors assembled with all three PAM-modified MPCs was improved. As the dosage increased from 0.5% to 2.0%, the electrochemical performance of APAM-CSSC and CPAM-CSSC increased initially and then decreased, while that of NPAM-CSSC showed continuous deterioration.

(6) APAM with a dosage of 0.5% had a low cost and comprehensively demonstrated an excellent synergy between mechanical and electrochemical properties. Compared to the unmodified MPC, it achieved an 88.6% increase in compressive strength (from 4.4 MPa to 8.3 MPa) while simultaneously delivering a 98.1% improvement in maximum areal capacitance (from 520 mF cm^−2^ to 1030 mF cm^−2^), corresponding to a 98.6% enhancement in areal energy density (from 0.072 mWh cm^−2^ to 0.143 mWh cm^−2^).

## Figures and Tables

**Table 1:** Mix proportions of PAM-MPC composites (g).

Mixture ID	MgO	KH_2_PO_4_	Na_2_B_4_O_7_·10H_2_O	PAM	H_2_O
NAPM-0.5%	100	100	10	1	120
NPAM-0.75%	100	100	10	1.5	120
NPAM-1.0%	100	100	10	2	120
NPAM-1.5%	100	100	10	3	120
NPAM-2.0%	100	100	10	4	120
CPAM-0.5%	100	100	10	1	120
CPAM-0.75%	100	100	10	1.5	120
CPAM-1.0%	100	100	10	2	120
CPAM-1.5%	100	100	10	3	120
CPAM-2.0%	100	100	10	4	120
APAM-0.5%	100	100	10	1	120
APAM-0.75%	100	100	10	1.5	120
APAM-1.0%	100	100	10	2	120
APAM-1.5%	100	100	10	3	120
APAM-2.0%	100	100	10	4	120

## Data Availability

The original contributions presented in this study are included in the article/Supplementary Materials. Further inquiries can be directed to the corresponding authors.

## Supplementary Materials

The following supporting information can be downloaded at: https://www.mdpi.com/article/10.3390/ma19071426/s1, Equation (S1): power density; Table S1: summary of FTIR absorption peaks and assignments.
