# Supplementary material for: Comparative Study on the Hydration, Mechanical Properties, and Energy Storage Performance of MPC-Based Solid Electrolytes Modified by Different Ionic PAMs
Source: Materials (Basel). 2026 Apr 2;19(7):1426. doi: 10.3390/ma19071426 (PMC13075171; doi:10.3390/ma19071426)
Supplement: Supplementary file 1 [file materials-19-01426-s001.zip › materials-4225226-supplementary.pdf]

materials MDPI

Supporting Information

Comparative Study on the Hydration, Mechanical Properties, and Energy Storage Performance of MPC-Based Solid Electrolytes Modified by Different Ionic PAMs

Jialu Liu [1], Yunpeng Zhang [2], Muyang Shi [1], Xin Shan [1,*] and Dong Zhang [1,*]

[1] Key Laboratory of Advanced Civil Engineering Materials, Ministry of Education, School of Materials Science and Engineering, Tongji University, Shanghai 201804, China; 2331576@tongji.edu.cn (J.L.); smyovo@126.com (M.S.)
[2] China Construction Eighth Engineering Division Co., Ltd., Shanghai 200112, China; zh_yunpeng@126.com
* Correspondence: shanxin@tongji.edu.cn (X.S.); zhangdng@tongji.edu.cn (D.Z.)

This PDF file includes:

Equation (S1)
The areal power density $P\alpha$ (mW cm^{-2}) of a symmetric CSSC is calculated from GCD curves using Equation (S1):

$$P_a = \frac{E_a \cdot 3600}{t} \quad \text{(S1)}$$

Where E_α is the areal energy density obtained from Equation (S1), and t is the discharge time (s).

Table S1 Summary of FTIR absorption peaks and assignments

Wavenumber (cm^{-1})	Peak feature	Assignment	Reference
430	sharp	Mg–O stretching vibration	[18, 34]
562	sharp	Mg–O vibration	[18, 34]
735	not sharp	PO_4^{3-} bending vibration (K-struvite)	[18, 34]
980	sharp	PO_4^{3-} antisymmetric stretching (K-struvite)	[18, 34]
1580	broadens with increasing PAM	Overlap region; becomes dominated by H-O–H bending (crystal water) + amide I (PAM)	[34–36]
1600–1700	emerging peak trend	Superposition: H-O–H bending (crystal water) + amide I band of PAM	[34–36]
2880	broad, not sharp	Masked C–H stretching (PAM amide groups) + downshifted O–H stretching (H_2O in K-struvite, strong hydrogen bonding)	[34, 36, 37]
